# Les péritonites postopératoires en réanimation: étude rétrospective, à propos de 46 cas

**DOI:** 10.11604/pamj.2022.43.125.14331

**Published:** 2022-11-04

**Authors:** Said Benlamkaddem, Nawfal Houari, Abderrahim El Bouazzaoui, Brahim Boukatta, Hicham Sbai, Nabil Kanjaa

**Affiliations:** 1 Service de Réanimation Polyvalente A4, Centre Hospitalier Universitaire Hassan II, Fès, Maroc

**Keywords:** Péritonites postopératoires, réintervention, lâchage d´anastomose, Postoperative peritonitis, reoperation, loosening of the anastomotic sutures

## Abstract

Il s´agit d´une étude portant sur 46 malades hospitalisés en réanimation pour péritonite postopératoire. L´incidence des péritonites postopératoires (PPO) dans notre travail était de 2,7%. L´âge moyen des malades était de 53,3 ans avec un sexe ratio de 1,2. La chirurgie sous-mésocolique était la plus pourvoyeuse de PPO (65,2%), dominé par la région colorectale (48%). Les signes cliniques étaient dominés par la fièvre (78%), les douleurs abdominales (57%), les signes extra-abdominaux. Le délai diagnostic était de 7,3 jours. La décision de la reprise chirurgicale sur les seuls critères cliniques et biologiques était la règle dans 56,5% des cas. La prise en charge thérapeutique était basée sur une réanimation périopératoire, un traitement des défaillances d´organes, une antibiothérapie probabiliste et une chirurgie par laparotomie médiane. Le profil bactériologique était dominé par les BGN (79%). Le lâchage d´anastomose était la cause directe de la PPO dans 57%. Le taux de mortalité était de 60%. Les principaux facteurs pronostiques étaient: l´insuffisance rénale, le nombre de défaillance viscérale, un TP < 50% la nécessite de ventilation et le recours aux catécholamines.

## Abstract (en)

We conducted a study of 46 patients admitted to the Intensive Care Unit with postoperative peritonitis. The incidence of postoperative peritonitis in this setting was 2.7%. The average age of patients was 53.3 years, with a sex ratio of 1.2. Submesocolic surgery was the most common cause of postoperative peritonitis (65.2%), mainly occurring in the colorectal region (48%). Clinical signs were dominated by fever (78%), abdominal pain (57%) and extra-abdominal signs. The mean time between symptom onset and diagnosis was 7.3 days. Reoperation was performed on the basis of clinical and biological criteria in 56.5% of cases. Therapeutic management was based on perioperative reanimation, treatment of organ dysfunctions, probabilistic antibiotic therapy and middle laparotomy. The bacteriological profile was dominated by BGN (79%). The loosening of the anastomotic sutures was the direct cause of PPO in 57% of patients. Mortality rate was 60%. The main prognostic factors were: renal failure, the number of patients with organ failure, PT<50%, the need for ventilation and the use of catecholamines.

## Introduction

Les péritonites postopératoires (PPO) constituent une complication grave de la chirurgie abdominopelvienne. C´est une urgence médico-chirurgicale, dont le pronostic dépend, de la rapidité et la qualité de prise en charge, du terrain sous-jacent et de l´étiologie.

## Méthodes

**Type de l´étude:** c´est une étude descriptive rétrospective monocentrique, qui avait inclus tous les patients présentant une péritonite postopératoire retenue sur des critères cliniques et/ou biologiques et/ou radiologiques hospitalisés en réanimation durant la période entre janvier 2010 et décembre 2011.

**Collecte des données:** les données ont été recueillies à partir des dossiers des patients à l´aide d´une fiche d´exploitation comportant les données démographiques, cliniques, biologiques, radiologiques, thérapeutiques et évolutives de chaque patient.

**L´analyse des données:** l´analyse des données a été réalisé à l´aide d´un logiciel SPSS20 dans le laboratoire d´épidémiologie de la faculté de médecine de Fès. Les résultats ont été exprimés en pourcentage pour les variables qualitatives et en moyenne pour les variables quantitatives. L'analyse univariée a été effectuée à l'aide du test Chi^2^ de Pearson avec un seuil de significativité p<0,05.

## Résultats

**Figure 1:**
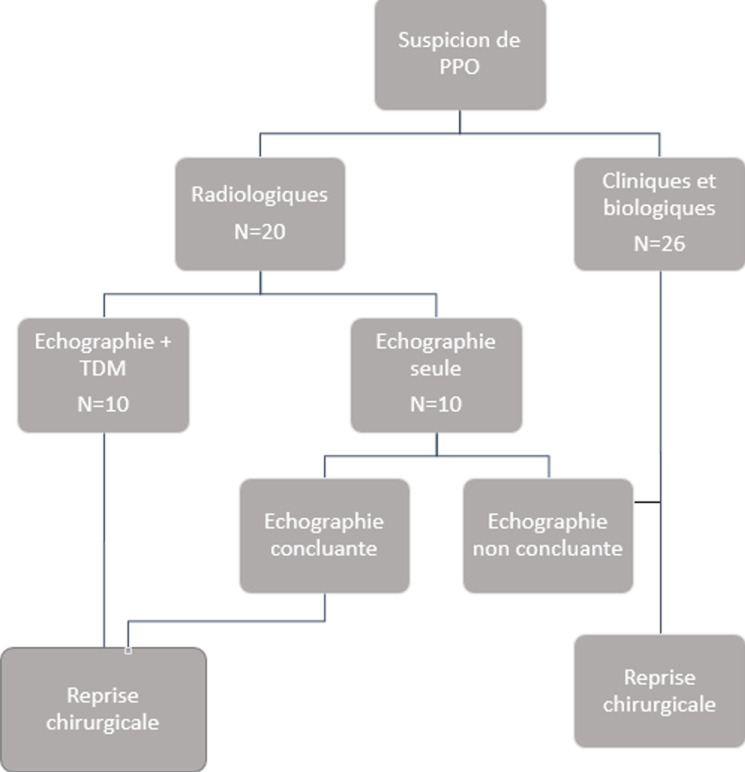
critères de réintervention dans notre étude

L´incidence des PPO dans notre travail durant la période d´étude, était de 2,7% parmi les laparotomies et 84% parmi les réinterventions. L´âge moyen des malades était de 53,3 ans avec un sexe ratio de 1,2 (25H/21F). Les caractéristiques des patients et de l´intervention initiale sont résumées dans, respectivement, les Tableau 1 et Tableau 2. Les signes cliniques étaient dominés par la fièvre (78%), et les signes extra-abdominaux notamment hémodynamiques (Tableau 3, Tableau 4). Le délai diagnostic était de 7,3 jours. La décision de la reprise chirurgicale sur les seuls critères cliniques et biologiques était la règle dans 67% des cas, alors que les critères radiologiques étaient à la base de l´indication chirurgicale dans 33% des cas (Tableau 5, Figure 1). La prise en charge thérapeutique était basée sur une réanimation péri opératoire, un traitement des défaillances d´organes, une antibiothérapie probabiliste et une chirurgie par laparotomie médiane.

**Tableau 1:** caractéristiques liées au terrain

Antécédent	Age (years)	Sex	Clinical presentation	Duration of symptoms (months)	Spinal region	Histological type	Preop Frankel grading	Postop Frankel grading
ASA > 2	48	F	Pain, Motor deficit	4	Thoracic	Psammomatous	D	D
Diabète	51	F	Motor deficit	24	Cervical	Angiomatous	B	D
Maladie CV	26	F	Pain, Motor deficit	2	Cervico-thoracic	Fibroblastic	C	C
Tabagisme	30	F	Pain, Motor deficit	12	Thoracic	Psammomatous	A	E
MICI	65	F	Motor deficit	36	Cervical	Psammomatous	C	D
Néoplasie	43	F	Motor deficit	12	Thoracic	Meningothelia	B	D
Radiothérapie	62	F	Pain, Motor deficit	18	Cervico-thoracic	Transitional	C	E
Chimiothérapie	28	F	Pain, Motor deficit	5	Thoracic	Transitional	A	A
Corticothérapie	55	F	Pain, Motor deficit	14	Cervico-thoracic	Meningothelia	B	B
Dénutrition	28	F	Pain, Motor deficit	60	Thoracic	Fibroblastic	B	D
Antibiothérapie préalable	45	F	Pain, Motor deficit	5	Thoracic	Meningothelia	C	D

**Tableau 2:** caractéristiques de l´intervention initiale

		% (N)
**Etage sus-mésocolique 35% (N=16)**	Estomac	15(7)
	Pancréas	9(4)
	Foie	4,5(2)
	VB	6,5(3)
**Etage sous mésocolique 65% (N=30)**	Rectum	24(11)
	Colon	24(11)
	Intestin grêle	6,5(3)
	Appendice	10,5(5)
**Chirurgie > 2heures**		72(33)
**Lieu de l´intervention initiale**	Urgences	43(20)
	Bloc central	57(26)
**Caractère septique**		31(14)
**Présence d´une anastomose digestive**		50(23)

**Tableau 3:** les signes abdominaux retrouvés chez nos patients

Signes cliniques	% (N)
Douleur abdominale	54(25)
Stase gastrique	37(17)
Arrêt des matières et des gaz	26(12)
Ballonnement abdominal	33(15)
Issue de liquide anormal	35(14)
Diarrhée	2(1)

**Tableau 4:** signes extradigestifs retrouvés chez nos patients

Signes extradigestifs	% (N)
Fièvre	74(34)
Hémodynamiques	70(32)
Respiratoires	58(27)
Neurologiques	28(13)
Insuffisance rénale	35(16)
Troubles hématologiques	26(12)
Défaillance multiviscérale	52(24)

**Tableau 5:** critères de réintervention

Décision de la reprise chirurgicale sur les seuls critères cliniques et biologiques
Choc septique (19 patients) Tableau franc de péritonite généralisée Issue de liquide anormal à travers les orifices de drainage ou les plaies opératoires. Eviscération
Décision de la reprise chirurgicale sur des critères radiologiques
Etat hémodynamique stable (13 patients) 2 patients étaient en choc septique mis sur le compte d´une pneumopathie grave et qui ont bénéficié en plus du scanner thoracique d’un scanner abdominal.

**Figure 2:**
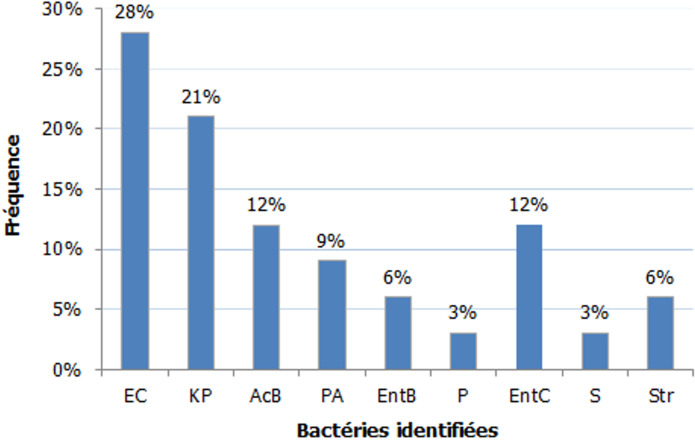
profil bactériologique des PPO dans notre travail

Les prélèvements bactériologiques réalisé en peropératoire ont permis d´avoir le profil bactériologique suivant (Figure 2): prédominance des BGN (79%) dominées par E. Coli (28%) suivie du *Klebsiella pneumoniae* (21%), *Acinetobacter baumanii* et l´entérocoque (12%). Le caractère multimicrobien était retrouvé dans 55%. L´association *E. Coli*-*Klebsiella pneumoniae* était la plus fréquente (37%). La ventilation mécanique et le recours aux amines étaient nécessaires dans respectivement 65% et 39%. Le lâchage d´anastomose était la cause directe de la PPO la plus fréquente (57%) (Tableau 6). La durée d´hospitalisation moyenne était de 8 jours. Le taux de mortalité était de 60%. Aucun paramètre lié au terrain ou à la chirurgie, n´a été statistiquement associé à la mortalité dans notre série. Les principaux facteurs pronostiques ressortis dans notre étude en analyse univariée, étaient: l´insuffisance rénale, le nombre de défaillance viscérale, un TP<50%, l´infection à *Acinetobacter Baumanii*, la nécessite de ventilation et le recours aux catécholamines (Tableau 7).

**Tableau 6:** causes directes de PPO retrouvées en peropératoire

Cause directe de la PPO	%
Lâchage	57
Perforations	24
Nécrose	14
Abcès	5

**Tableau 7:** facteurs pronostiques des PPO dans notre travail

		Mortalité(%)	P
IR		67,8	0,032
Défaillance viscérale	0	0	0,006
	1	40	
	2	67	
	≥3	100	
Infection à AcB		100	0,02
VM		81	<0,05
Catécholamines		83,3	<0,05

## Discussion

Les péritonites postopératoires constituent une complication relativement rare mais très grave de la chirurgie abdominopelvienne, son incidence est variable en fonction des études (3% en moyenne) [1-3], les facteurs de risque sont multiples, liés au terrain (sexe masculin, dénutrition, néoplasie, immunodépression, dénutrition, tares associé, maladies inflammatoires de l´intestin, radiothérapie, un score ASA élevé…) [4-10] ou lié à l´intervention initiale (caractère urgent, contexte septique, durée longue, site sous-mésocolique, transfusion peropératoire, expérience du chirurgien….) [7,10-12]. La fièvre est le signe le plus fréquent [13], surtout, si elle survienne entre le troisième et le dixième jour postopératoire [14]. Les signes abdominaux sont difficiles à interpréter dans ce contexte et les signes extra-abdominaux, fréquents, peuvent orienter à tort vers une pathologie extra-abdominale [12].

**Figure 3:**
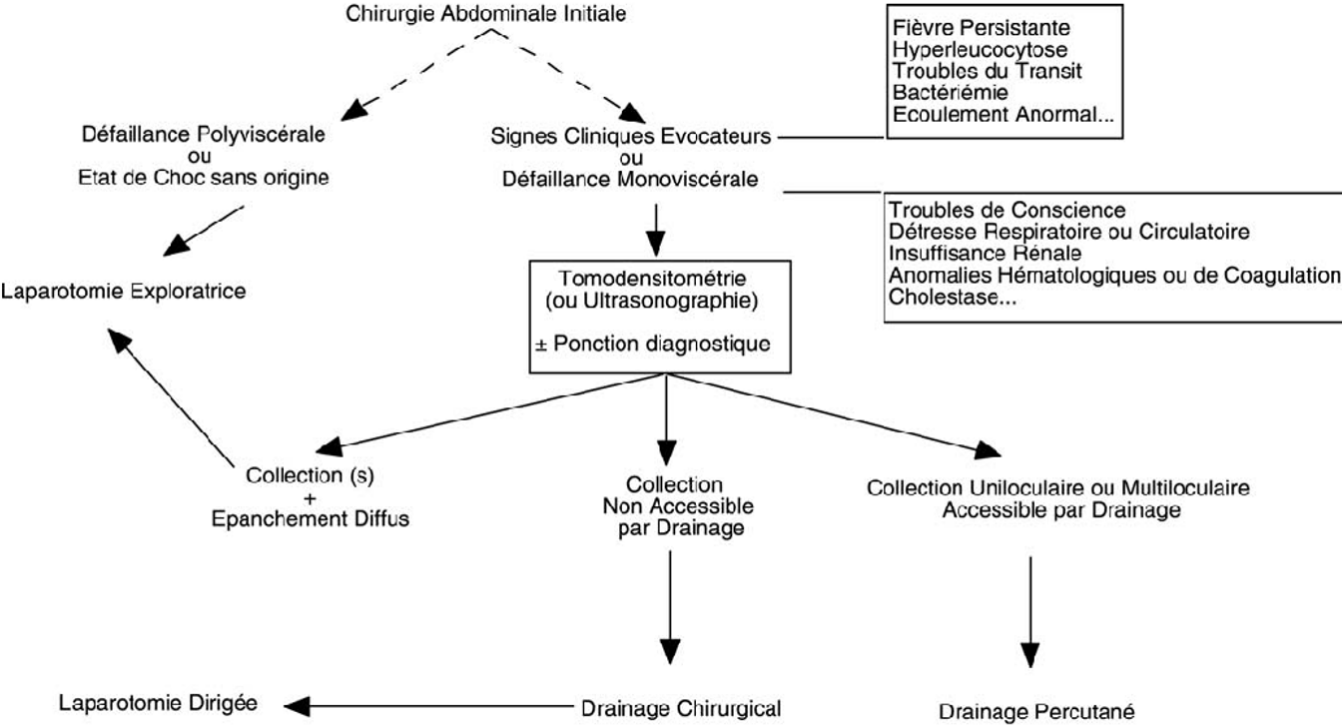
arbre décisionnel en cas d´évolution anormale au décours d´une chirurgie abdominale

Le diagnostic doit être évoqué de principe, devant toute évolution anormale en postopératoire [12]. La rapidité avec laquelle le diagnostic est porté et l´efficacité du traitement mis en œuvre conditionnent le pronostic. Pour Koperna et Schulz, seule une décision de ré-exploration rapide au cours des 48 premières heures suivant le diagnostic permet de diminuer la mortalité [15]. Bohnen *et al*. rapportent une mortalité de 35% en cas de réintervention précoce contre 65% en cas de réintervention plus tardive [16]. De ce fait, la survenue de défaillances polyviscérales ou l´apparition d´un état de choc sans origine évidente seront des critères formels de réintervention. Une laparotomie blanche est toujours moins grave par rapport à une reprise chirurgicale tardive En dehors de cette situation, la décision de reprise chirurgicale se basera sur un faisceau d´arguments cliniques et biologiques étayés par les données morphologiques (Figure 3) [17,18].

La prise en charge thérapeutique est multidisciplinaire impliquent anesthésiste réanimateur, chirurgien, radiologue et microbiologiste. Elle comporte une réanimation hémodynamique rapide et optimale, une antibiothérapie probabiliste choisie en fonction du profil bactériologique de la structure hospitalière et du caractère nosocomiale de l´infection et adaptée à l´antibiogramme, et un geste chirurgical le plus parfait possible. La mortalité est variable en fonction des études, entre 35 et 60% [5,11,12]. Plusieurs facteurs pronostic ont été rapportée (l´âge avancé, les comorbidités, nombre de défaillance d´organe, caractère adapté ou non de l´antibiothérapie, type de la chirurgie initiale, délai de prise en charge…) [12,15,18,19].

## Conclusion

Les péritonites postopératoires constituent une complication grave de la chirurgie abdominale, de diagnostic souvent difficile. La prise en charge repose sur une approche multidisciplinaire dans laquelle l´anesthésiste réanimateur joue un rôle central. Seule une gestion thérapeutique efficace et précoce permet de réduire la mortalité qui reste encore élevée durant ces dernières années malgré les différents progrès réalisés dans le domaine de chirurgie et de réanimation.

### Etat des connaissances sur le sujet

- Les péritonites postopératoires constituent une urgence médicochirurgicale dont la rapidité de prise en charge est un facteur qui conditionne le pronostic;
- Le diagnostic est difficile en période postopératoire et nécessite une collaboration multidisciplinaire;
- Une laparotomie blanche est mieux qu´un sepsis intrapéritonéal non traité.

### Contribution de notre étude à la connaissance

- Partager notre expérience sur les PPO;
- Comparer notre attitude diagnostique et thérapeutique avec la littérature;
- Relever les facteurs de mortalité propre à notre contexte.
